# Temporomandibular Joint Abnormalities in Hemodialysis Patients: A Cross-Sectional Ultrasonographic Study from a Single Center in Italy

**DOI:** 10.3390/medsci14040492

**Published:** 2026-08-19

**Authors:** Beatrice Maranini, Andrea Brunati, Marcello Govoni, Stefano Mandrioli, Manlio Galiè, Fabio Fabbian

**Affiliations:** 1 Department of Medical Sciences, University of Ferrara, I-44121 Ferrara, Italy; beatrice.maranini@edu.unife.it (B.M.); andrea.brunati@edu.unife.it (A.B.); marcello.govoni@unife.it (M.G.); 2 Department of Cranio-Maxillofacial Surgery, Unit of Cranio-Maxillofacial Surgery, University Hospital of Ferrara, I-44124 Ferrara, Italy; s.mandrioli@ospfe.it (S.M.); m.galie@ospfe.it (M.G.)

**Keywords:** temporomandibular joint, ultrasound, hemodialysis, chronic kidney disease, renal osteodystrophy

## Abstract

**Background/Objectives:** Temporomandibular joint (TMJ) abnormalities are a common finding in the general adult population, but they have been rarely investigated in people receiving chronic dialysis. The TMJ is a unique synovial joint that can be altered by either inflammatory or degenerative processes, both of which are amenable to ultrasound (US) assessment. The aim of this study was to describe the pattern of TMJ involvement in a cohort of hemodialysis patients using TMJ ultrasound (TMJ US). **Methods:** This cross-sectional, single-center study evaluated the clinical utility of TMJ US to detect inflammatory and degenerative changes in patients undergoing chronic hemodialysis and was carried out between June and December 2025. Demographic data, dialysis vintage, the Controlling Nutritional Status (CONUT) score and the Charlson Comorbidity Index (CCI) were collected and related to TMJ US findings and to bone-metabolism biomarkers (calcium, phosphate, parathyroid hormone). **Results:** 100 hemodialysis patients were included (65 male, 65%; mean age 71.6 ± 13.1 years). Comorbidity and undernutrition were frequent findings (CCI ≥ 4 in 63%; CONUT ≥ 3 in 68%). TMJ US revealed that degenerative indicators were more common than inflammatory ones: calcifications (36%), condylar irregularities (33%) and enthesophytes (25%) were the most prevalent degenerative findings, whereas joint effusion (16%), synovial hypertrophy (15%) and cartilage changes (15%) were the leading inflammatory findings; a positive power Doppler signal was rare (1%). Cortical/condylar irregularity was significantly more prevalent in patients with a dialysis vintage of less than 30 months (43.8% vs. 23.1%, *p* = 0.034), who also had lower serum calcium levels. No other TMJ US finding showed a significant association with age, sex, comorbidity burden, nutritional status, or bone-metabolism parameters on univariate. **Conclusions:** TMJ US demonstrated a substantial burden of subclinical degenerative and, to a lesser extent, inflammatory TMJ findings in this cohort of hemodialysis patients; none of these abnormalities were spontaneously reported as symptomatic, and their prevalence was independent of age, dialysis vintage, comorbidity, nutritional status and classical bone-metabolism parameters. These findings cannot be directly attributed to the dialysis/uremic environment rather than to age-related degeneration; they support the concept that the TMJ warrants further investigation as a potentially under-recognized target within the broader systemic metabolic derangement of end-stage kidney disease, and suggest that TMJ US is a feasible, accessible technique for detecting subclinical TMJ involvement in this population, pending confirmation of clinical utility in controlled studies.

## 1. Introduction

Temporomandibular joint (TMJ) disorder encompasses a heterogeneous group of conditions involving pain in the jaw joint and associated musculature, restricted jaw movement, or joint clicking during function [1]. TMJ abnormalities are common and typically self-limiting; epidemiological data indicates a high clinical prevalence, with up to 75% of the general population displaying physical signs on examination, contrasted by a substantially lower symptomatic prevalence of roughly 33% [2,3].

The TMJ is a unique synovial joint featuring an articular disc that allows both hinge and gliding movements, which are essential for chewing and speech. Unlike most synovial joints, its articular surfaces are covered by fibrous connective tissue and only a thin layer of hyaline cartilage, which makes TMJ prone to sudden damage and erosion if TMJ disorder is not early detected [4,5].

The etiology of TMJ abnormalities is considered multifactorial, involving capsular inflammation, muscle spasm and disc abnormalities [6]. Although stress, anxiety and parafunctional habits such as bruxism have traditionally been implicated, more recent evidence challenges some of these associations: malocclusion is found with similar frequency in healthy individuals and in those with TMJ abnormalities, and its correction rarely resolves symptoms [7,8,9,10]. Articular abnormalities of the TMJ span a wide spectrum, including ankylosis, congenital or developmental anomalies, neoplasia, internal disc derangement with or without reduction, condylar fractures, inflammatory disorders (synovitis, capsulitis, polyarthritis), osteoarthritis and dislocation [6,11]. Moreover, as a synovial joint, the TMJ is susceptible to the same rheumatologic pathology that affects any other joint in the body [12]. This narrow framing can lead clinicians to overlook systematic TMJ screening in patients with systemic rheumatic disease [13]. TMJ ultrasound (TMJ US) has been studied as a reliable tool to distinguish inflammatory from degenerative TMJ changes across different patient populations, including rheumatic and non-rheumatic cohorts, offering a radiation-free, dynamic and widely available alternative to magnetic resonance imaging (MRI), which still remains the imaging gold standard, for first-line assessment [14,15].

Bone and kidney physiology are intrinsically linked through a multifactorial regulatory network often described as the “kidney–bone axis”, in which skeletal health is closely tied to renal function [16]. Proper bone metabolism depends on the kidney’s capacity to activate vitamin D (via 1-hydroxylase), produce erythropoietin, and clear or modulate signaling proteins such as fibroblast growth factor 23 (FGF23) [17,18]. Derangement of this axis becomes most clinically evident in renal osteodystrophy and in chronic kidney disease-mineral and bone disorder (CKD-MBD) [19,20]. A 2025 KDIGO Controversies Conference reaffirmed that CKD-MBD remains a systemic syndrome integrating biochemical, skeletal and vascular abnormalities, while proposing a refined conceptual framework distinguishing CKD-associated osteoporosis from CKD-associated cardiovascular disease for future guideline development [21], and recent clinical evidence in patients with CKD shows that the FGF23-Klotho axis directly promotes renal tubular expression of inflammatory cytokines, supporting a role for immune-mediated mechanisms in these derangements [22]. Specific craniofacial and jaw changes may represent early clinical markers of CKD-MBD at different disease stages [23].

Despite all these considerations, investigations specifically addressing TMJ abnormalities in uremic individuals remain scarce, and most available data derives from self-reported symptoms rather than objective imaging.

We therefore designed a cross-sectional study to describe through TMJ US the major anatomical alterations of the TMJ in a cohort of patients undergoing chronic hemodialysis, and to explore their relationship with demographic, nutritional, comorbidity and bone-metabolism parameters.

## 2. Materials and Methods

### 2.1. Study Population

This cross-sectional observational study was conducted between June and December 2025, enrolling patients with end-stage renal disease (ESRD) receiving maintenance hemodialysis at the Dialysis Unit of the University Hospital of Ferrara, Italy, a center that chronically treats approximately 136 patients per year. Given the descriptive aim of the study, all patients meeting the inclusion criteria were consecutively offered participation unless they met an exclusion criterion or declined to take part.

No a priori sample size calculation was performed, since the study was designed as a hypothesis-generating, descriptive survey of TMJ US findings rather than a hypothesis-testing comparison; the target enrolment of approximately 100 patients was set pragmatically to capture roughly one six-month cohort of consecutively treated patients at the center, consistent with similar single-center descriptive ultrasonographic studies in this population.

Inclusion criteria were:

- Age ≥ 18 years;
- Diagnosis of ESRD;
- Hemodialysis treatment for more than one month.

Exclusion criteria were:

- Age < 18 years;
- Previous TMJ surgery;
- Acute TMJ trauma;
- Active infection;
- Inability to undergo ultrasound;
- Incomplete clinical data.

To evaluate TMJ US abnormalities of the hemodialysis group, data from 40 healthy controls and from 46 patients belonging to the rheumatological clinic were compared. However, the healthy control group was considerably younger and had a different sex distribution than our hemodialysis cohort, because of this, the healthy controls were not considered representative of the general healthy population, but we used their data only as supportive information. Moreover, an age-comparable rheumatology outpatient cohort was evaluated with the aim of obtaining additional contextual information about TMJ abnormalities.

**Figure 1:**
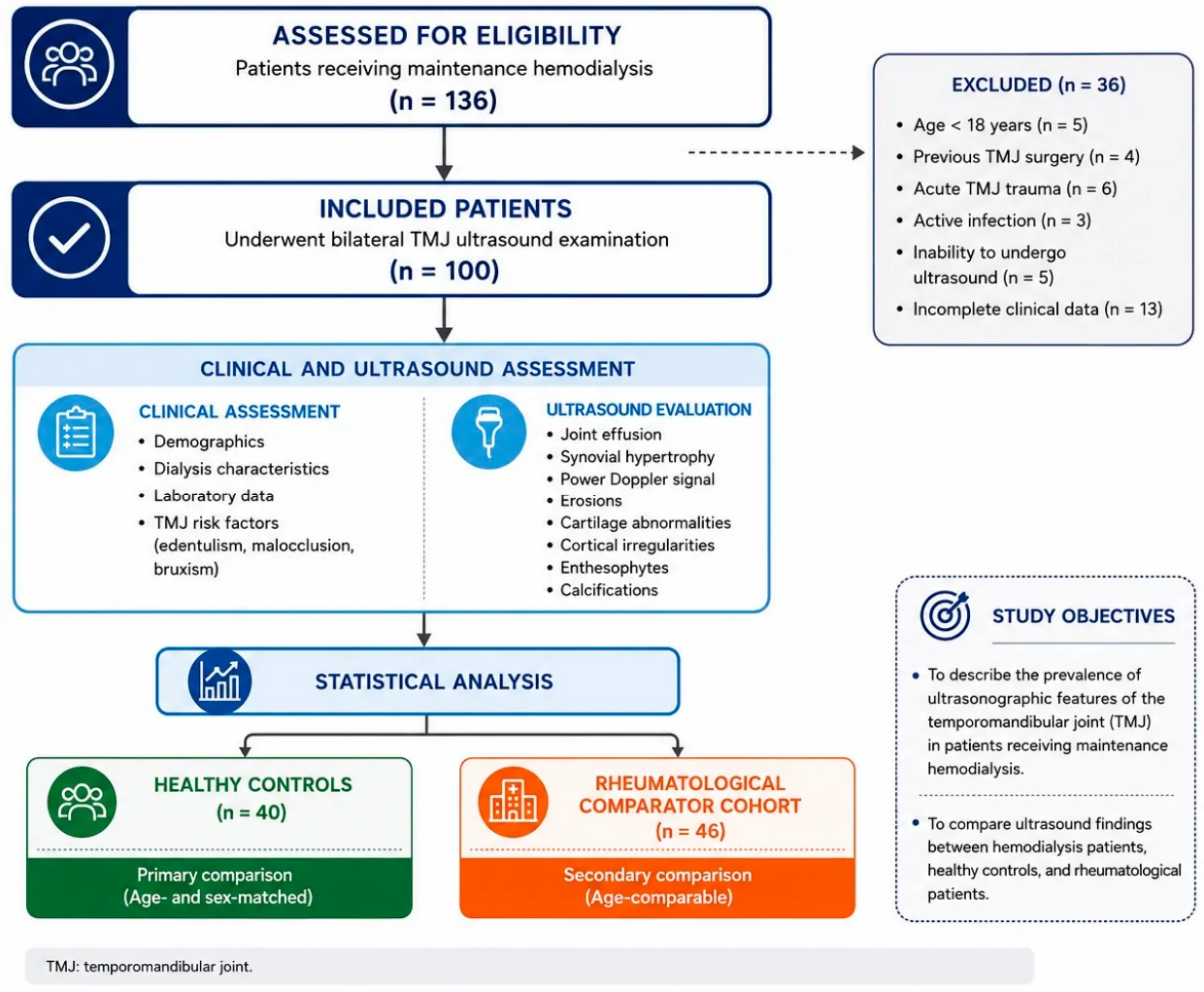
Graphical study flow illustrating patient inclusion and exclusion.

The flow diagram illustrating patient inclusion is reported in Figure 1.

The primary outcome of the study was the prevalence and pattern of ultrasonographic temporomandibular joint abnormalities, including both inflammatory findings (joint effusion, synovial hypertrophy, Power Doppler signal and cartilage abnormalities) and structural changes (cortical irregularities, erosions, enthesophytes, and calcifications), in patients undergoing chronic hemodialysis. Secondary outcomes included the evaluation of possible associations between TMJ ultrasonographic abnormalities and demographic characteristics, laboratory parameters, and dialysis-related variables.

### 2.2. Data Collection

Demographic data (age, sex) and dialysis vintage (months on hemodialysis) were recorded for each participant. Nutritional status was assessed using the Controlling Nutritional Status (CONUT) score, a validated screening tool that combines three routinely available laboratory parameters (serum albumin, total cholesterol and total lymphocyte count) into a single composite score. Beyond its role as a nutritional screening instrument, a high CONUT score is a recognized prognostic marker, with higher scores associated with adverse outcomes including increased mortality in patients with chronic kidney disease [24,25].

Comorbidity burden was quantified using the Charlson Comorbidity Index (CCI), a validated tool that assigns weighted points to specific conditions according to their impact on survival, generating a cumulative score reflecting overall comorbidity burden [26]. The original index was adapted for this study by removing the diagnosis of renal disease, since all participants were, by definition, affected by ESRD [27].

All patients underwent a standardized clinical evaluation of the TMJ: the assessment included the presence of TMJ pain at rest or during mandibular movements, joint sounds (clicking or crepitus), mandibular range of motion, and tenderness on palpation. None of the patients reported TMJ-related symptoms or showed clinical findings suggestive of temporomandibular abnormalities at the time of the examination. For this reason, no validated TMD questionnaire was administered, as this was beyond the scope of the study.

### 2.3. Ultrasonographic Study of the Temporomandibular Joint

TMJ US examinations were performed by a single rheumatologist with more than five years of dedicated experience in musculoskeletal and TMJ ultrasound, to minimize inter-observer variability. Patients were examined in the supine position, and a high-frequency linear transducer (12–18 MHz; Esaote MyLab Sigma) was positioned parallel to the mandibular condyle and perpendicular to the zygomatic arch, in keeping with previously described and validated scanning protocols [14,28,29,30]. Both the right and left joints were systematically assessed for the following pathological markers. Sonographic findings were defined, in accordance with previously validated criteria [14,28,29,30], as follows:

- Joint effusion, anechoic-to-hypoechoic, non-compressible intra-articular fluid distending the joint capsule;
- Synovial hypertrophy, hypo-/iso-echoic, non-displaceable intra-articular tissue with or without an associated power Doppler signal;
- Cartilage changes, thinning or an irregular, heterogeneous echotexture of the fibrocartilaginous surface;
- Condylar/cortical irregularities, loss of the normal smooth hyperechoic cortical contour of the condyle;
- Enthesophytes, hyperechoic bony spurs at ligament/capsule bone insertions; and calcifications, hyperechoic foci within the joint or periarticular soft tissue, with or without posterior acoustic shadowing.

For each finding, a patient was classified as positive if the abnormality was present in at least one of the two joints (right or left); bilateral versus unilateral involvement was recorded but not analyzed as a separate outcome in the present dataset. All examinations were performed by a single experienced rheumatologist and the ultrasonographer was blinded both to laboratory data and the patients’ clinical status.

US features were distinguished in:

(A) Inflammatory findings (active/acute joint distress)

Joint effusion; synovial hypertrophy; power Doppler (PD) signal indicating increased intra-articular blood flow (hyperemia); and cartilage changes, defined as thinning or an irregular, heterogeneous echotexture of the fibrocartilaginous surface.

(B) Degenerative findings (chronic structural change)

Structural abnormalities of the condylar profile; condylar/cortical irregularities; enthesophytes; erosions; and calcifications.

Deeper definitions of US variables are reported in Supplementary Table S1.

The examination protocol and the sonographic definitions of each pathological finding were based on previously published TMJ ultrasonography studies and validated scanning methodology [29,30]. All US examinations were performed by a single rheumatologist with extensive experience in musculoskeletal and TMJ ultrasonography, thereby minimizing inter-observer variability.

### 2.4. Blood Sampling and Laboratory Tests

Laboratory data were retrieved from the electronic clinical database of the University Hospital of Ferrara and derived from routine follow-up testing performed by the hospital’s automated laboratory analyzer. Mean values of serum calcium and phosphate over the six months preceding the TMJ US examination and mean parathyroid hormone (PTH) values over the preceding twelve months, were used. Mean values of albumin, total cholesterol and lymphocyte count over the six months preceding the study were used to calculate the CONUT score.

### 2.5. Ethics

The study was conducted in accordance with the Declaration of Helsinki and was approved by the Institutional Research Board of the University Hospital of Ferrara (approval number: CET-AVEC 590-2024-Oss-AOUFe, approved at 17 October 2024). All participants received a full explanation of the study procedures and provided written informed consent prior to enrolment.

### 2.6. Statistical Analysis

Descriptive statistics were used throughout. Categorical variables were expressed as absolute and relative frequencies; continuous variables were expressed as mean ± standard deviation (SD). Serum calcium, serum phosphate and PTH were log-transformed (natural logarithm) prior to analysis to approximate a normal distribution. The cohort was dichotomized by age (median, 73 years) and by dialysis vintage (median, 30 months). Between-group comparisons used the independent-samples *t*-test or the Mann-Whitney U test for continuous variables, as appropriate, and the χ^2^ test or Fisher’s exact test for categorical variables. Multiple comparisons and dichotomization of continuous variable such as age and hemodialysis vintage using their median value, were used to better define the impact of the two parameters on TMJ US findings. A two-sided *p* value < 0.05 was considered statistically significant; given the exploratory, hypothesis-generating nature of this descriptive study and the number of comparisons performed, *p* values were not adjusted for multiplicity and should be interpreted with this in mind. All analyses were performed using IBM SPSS Statistics version 24.0 (IBM Corp., Armonk, NY, USA).

## 3. Results

**Figure 2:**
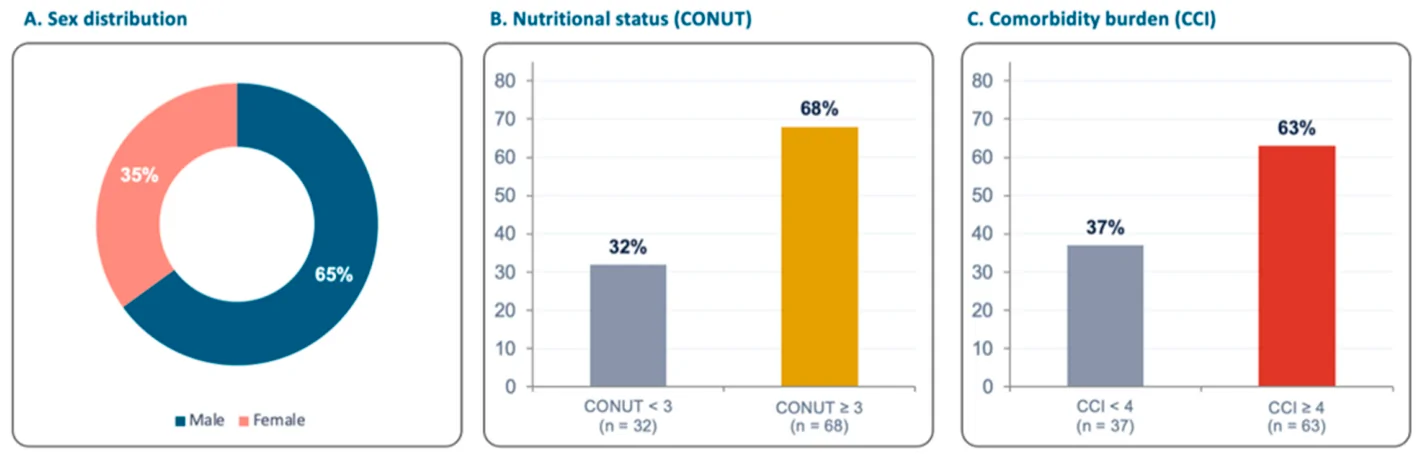
Demographic, nutritional, and comorbidity profile of the study cohort (Panel (**A**): sex distribution; Panel (**B**): nutritional status by CONUT category; Panel (**C**): comorbidity burden by CCI threshold).

Of 136 patients chronically treated at the center during the study period, 100 hemodialysis patients met the inclusion criteria and were enrolled (65 males, 65%; 35 females, 35%). The median age of the population was 73 years, and the median hemodialysis vintage was 30 months. The CCI (median value 4) was ≥4 in 63 patients (63%), and the CONUT score (median value 3) was ≥3 in 68 patients (68%) and ≥4 in 37 patients (37%), confirming a high burden of comorbidity and undernutrition in this population. Mean values of demographic, laboratory and clinical parameters are summarized in Table 1 and Figure 2.

**Figure 3:**
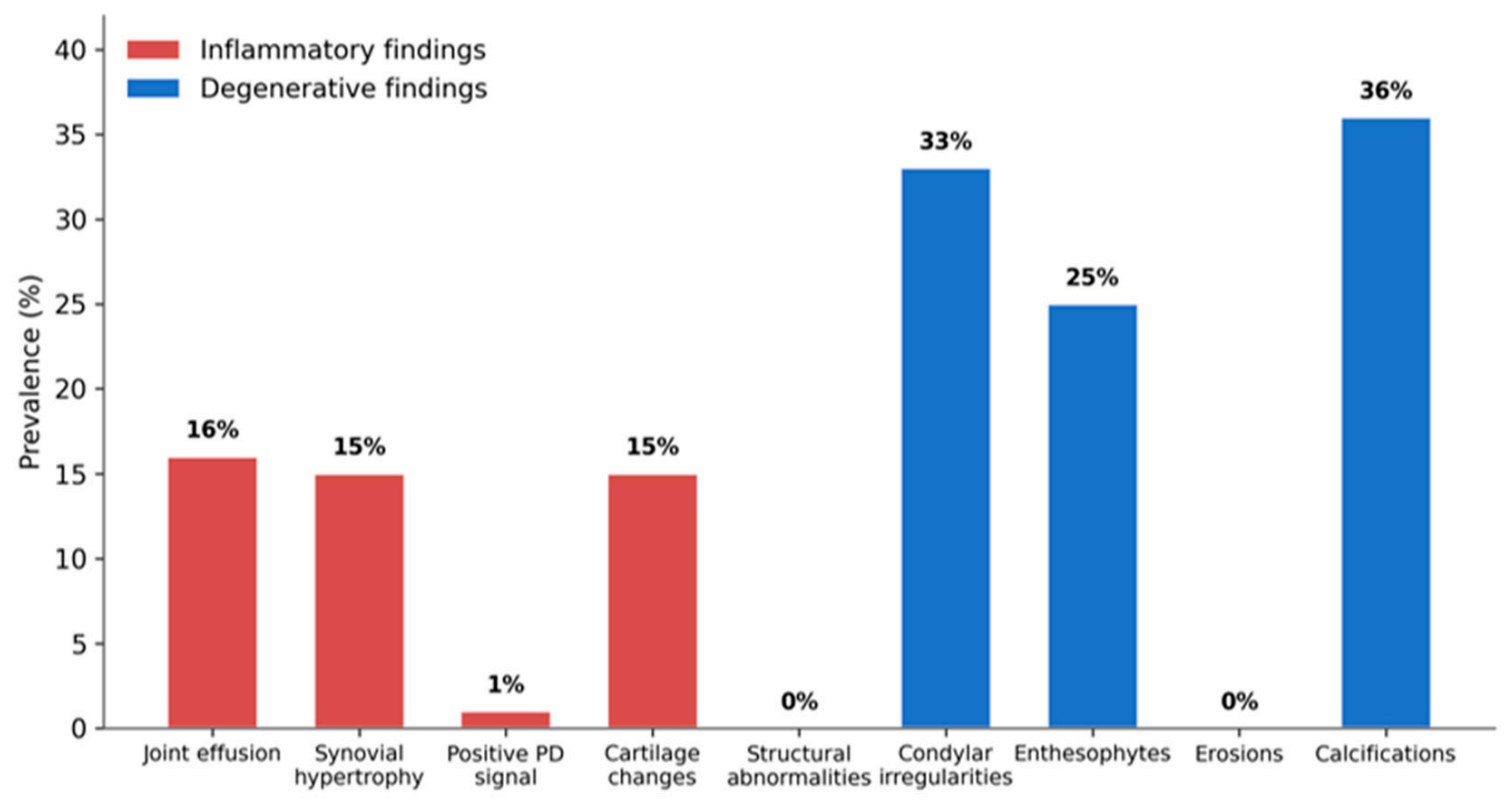
Prevalence of ultrasonographic TMJ findings in the overall cohort of 100 hemodialysis patients, grouped by inflammatory (red) and degenerative (blue) patterns.

TMJ US data for the enrolled subjects are reported in Table 2 and Figure 3. Regarding inflammatory indicators, joint effusion was observed in 16 patients (16%), synovial hypertrophy in 15 (15%), a positive Doppler signal in only 1 (1%), and cartilage changes in 15 (15%). Regarding degenerative and structural indicators, no patient showed frank structural abnormalities (0%) or erosions (0%), while condylar irregularities were present in 33 patients (33%), enthesophytes in 25 (25%), and calcifications in 36 (36%). Overall, TMJ US findings indicated that degenerative indicators were considerably more common than inflammatory ones.

The distribution of unilateral and bilateral TMJ abnormalities is summarized in Supplementary Table S2. Most abnormalities were unilateral, whereas bilateral involvement was observed less frequently and mainly affected degenerative structural changes such as cortical irregularities and calcifications.

The prevalence of edentulism, malocclusion, and bruxism and their relationship with ultrasonographic TMJ findings are reported in Supplementary Table S3. None of these variables showed a significant association with the main ultrasonographic abnormalities.

To contextualize these findings against a non-uremic reference population, TMJ US data from the 100 hemodialysis patients were compared with those of 40 healthy controls drawn from the same multicenter ultrasonographic dataset. Compared with healthy controls, hemodialysis patients showed a significantly higher prevalence of joint effusion (16% vs. 2.5%, *p* = 0.041), synovial hypertrophy (15% vs. 2.5%, *p* = 0.040), condylar/cortical irregularities (33% vs. 2.5%, *p* < 0.0001), unilateral condylar/cortical irregularities (23% vs. 2.5%, *p* = 0.002), enthesophytes (25% vs. 0%, *p* = 0.0001; monolateral 24% vs. 0%, *p* = 0.0003), and unilateral calcifications (26% vs. 0%, *p* = 0.0001). No significant between-group differences emerged for erosions, power Doppler signal, reduced cartilage thickness, structural dishomogeneity, or disc dislocation (Table 3).

To provide additional context, we also compared the hemodialysis cohort with an independent age-comparable rheumatology outpatient cohort without clinically active rheumatic disease involving the TMJ (Supplementary Table S4). Although the two groups differed significantly in sex distribution, cortical irregularities and calcifications remained significantly more frequent in patients undergoing hemodialysis, whereas no significant differences were observed for inflammatory ultrasonographic findings.

**Figure 4:**
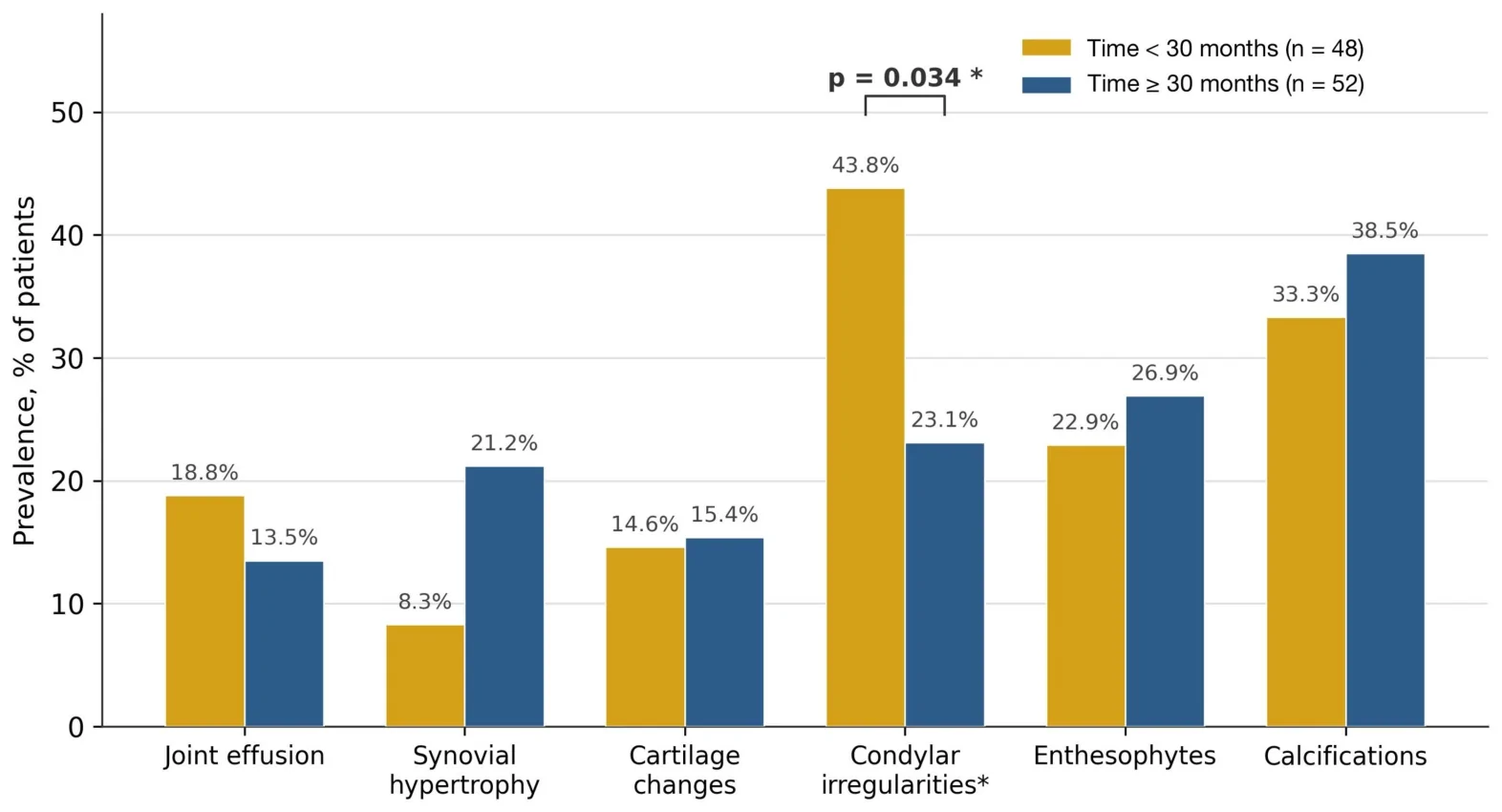
TMJ US findings stratified by hemodialysis time (<30 vs. ≥30 months). Condylar irregularities were significantly more prevalent in patients with a shorter dialysis vintage (* *p* = 0.034).

The study population was subsequently dichotomized according to the median age (73 years) and median dialysis vintage (30 months); results of these univariate analyses are detailed in Table 4 and Table 5 and Figure 4.

As expected, patients aged ≥ 73 years had a significantly higher CCI (5.22 ± 1.71 vs. 3.06 ± 1.64, *p* < 0.001) and a higher proportion with CCI ≥ 4 (90% vs. 36%, *p* < 0.0001), but no TMJ US finding differed significantly between age groups. The only TMJ US finding significantly associated with a dialysis-related parameter was a higher prevalence of condylar/cortical irregularity in patients with a dialysis vintage of less than 30 months (43.8% vs. 23.1%, *p* = 0.034); these patients also displayed significantly lower serum calcium levels (8.9 ± 0.5 vs. 9.2 ± 0.5 mg/dL, *p* = 0.046). 

## 4. Discussion

This study explores the pattern and prevalence of TMJ alterations in patients undergoing chronic hemodialysis.

To the best of our knowledge, this is the first study to systematically evaluate the TMJ through the US, which is an accessible, radiation-free technique capable of detecting inflammatory and degenerative change as well as calcification, in an adult hemodialysis population.

Although MRI remains the gold standard imaging modality for comprehensive evaluation of the temporomandibular joint, particularly for assessing the articular disc and early inflammatory changes, its routine use in asymptomatic patients undergoing chronic hemodialysis is limited by cost, availability, and examination time. In contrast, ultrasonography is a rapid, bedside, non-invasive and inexpensive imaging modality that is particularly suitable for detecting structural TMJ abnormalities in clinical practice. Moreover, the reliability of TMJ ultrasonography has previously been demonstrated by our group and by other published studies [29,30] supporting its use in observational studies such as the present one.

We categorized joint abnormalities into two primary groups:

(i) Inflammatory alterations, demonstrated by joint effusion, synovial hypertrophy, a positive power Doppler signal indicating increased blood flow and reduced articular cartilage thickness;
(ii) Degenerative alterations, confirmed by bone erosions, cortical irregularities, enthesophytes and calcifications.

We related these findings to demographics, age, dialysis vintage, comorbidity burden, nutritional status, and bone-metabolism parameters, but were unable to demonstrate a clear, consistent relationship. We could only confirm that degenerative indicators were more common than inflammatory ones, and that condylar/cortical irregularity was more prevalent among patients with a shorter dialysis vintage.

Joint problems in hemodialysis patients have been recognized since the 1980s, with reported patterns including periarticular calcification, carpal tunnel syndrome, avascular necrosis, tendon rupture, infection, and recurrent hemarthrosis progressing to chronic capsulitis [31]. The relationship between the TMJ and advanced chronic kidney disease (CKD) is complex, and the joint may be caught in the crossfire of the systemic metabolic derangements that accompany declining renal function [32].

Cunha et al. clinically evaluated the oral health of 160 Brazilian hemodialysis patients (mean age 59 ± 12 years), assessing TMJ status by clicking, tenderness on palpation, reduced mobility, pain or difficulty opening/closing the mouth. With dialysis vintage ranging from 11 months to 11 years, only 2.5% and 3.8% of participants reported signs and symptoms of TMJ disorder, respectively [33]. Bots et al. studied 42 Dutch patients with CKD (mean age 42.6 ± 9.2 years; 66.7% on hemodialysis, 33.3% on peritoneal dialysis) and reported self-reported TMJ complaints in 16.7% of cases [34]. In 2022, Yilmaz et al. reported a prevalence of TMJ disorder of 41.5% in a Turkish chronic hemodialysis population, with TMJ pain as the most frequent symptom; C-reactive protein, hemoglobin, PTH and albumin were significantly associated with TMJ disorder in that cohort [35]. In 2020, Somay et al. described a high prevalence of sleep bruxism and TMJ-related symptoms, including limited mouth opening, deviation/deflection, clicking, muscle tenderness, pain, among 68 hemodialysis patients [36]. More recently, Morozova et al. applied TMJ ultrasound screening to 40 children across different stages of CKD, including patients on hemodialysis and after renal transplantation, and found a high frequency of anterior disc displacement across all disease stages, supporting the use of US as an early, non-invasive adjunctive imaging technique for TMJ pathology across the CKD spectrum, from pediatric to adult populations [37].

Isolated case reports further illustrate the breadth of TMJ pathology described in dialysis populations: acute hydroxyapatite deposition periarthritis in a young woman on peritoneal dialysis [38], degenerative TMJ disease in a patient with Alport syndrome [39], and dialysis-related amyloidosis of the TMJ in a long-term hemodialysis patient [40]. Calcium, phosphate and PTH plausibly play a pivotal role in TMJ damage; earlier authors have suggested that macrognathia and altered jaw morphology may represent secondary complications of renal osteodystrophy that could in turn affect the TMJ [41,42].

Long-term hemodialysis frequently triggers a cascade of metabolic imbalances that compromise the structural integrity of tendons, ligaments and entheses. This degradation is thought to arise from three interconnected mechanisms:

(i) Secondary hyperparathyroidism, in which chronic excess PTH directly promotes biochemical degradation of tendon tissue;
(ii) Reduced active vitamin D and altered vitamin D receptor expression, causing denaturation of collagen fibers within ligaments and tendons;
(iii) Chronic metabolic acidosis, which impairs collagen synthesis and the replacement of damaged fibers.

The synergy of high PTH, low vitamin D activity and metabolic acidosis is thought to favor the formation of atypical collagen constituents [43].

The enthesis, the anatomical site where ligaments, tendons and joint capsules attach to bone, serves as a critical stress-transfer point between soft tissue and skeleton [44]. In hemodialysis patients, enthesitis is a recognized but frequently subclinical concern, and its true prevalence in the uremic population is likely underestimated [45]. A previous study of 49 hemodialysis patients using the Glasgow Ultrasound Enthesitis Scoring System (GUESS) demonstrated a direct relationship between dialysis duration and increased thickness of the quadriceps tendon, Achilles tendon and plantar fascia [46].

Interestingly, in our cohort we found no statistically significant association between TMJ abnormalities and overall hemodialysis duration, demographics, comorbidity, nutritional status, or bone-metabolism parameters, and no patient spontaneously reported TMJ symptoms. These findings suggest that overt clinical characteristics may not reliably act as biomarkers of TMJ derangement in this setting; nonetheless, as in previous studies, we confirm a considerable prevalence of TMJ abnormalities in end-stage kidney disease, here demonstrated objectively by ultrasound rather than by symptom report alone [35,47]. When directly compared with healthy controls, hemodialysis patients displayed a markedly higher burden of both inflammatory (effusion, synovial hypertrophy) and degenerative (cortical irregularities, enthesophytes, calcifications) TMJ US findings, supporting the hypothesis that these changes are not simply age-related but reflect the systemic metabolic derangement of end-stage kidney disease. This comparison should nonetheless be interpreted cautiously: the control group was substantially younger (mean age 51.4 vs. 71.7 years) and included a higher proportion of women, so age and sex confounding cannot be excluded, and formal age-/sex-adjusted analysis was not feasible within the scope of this study.

We also made comparisons with a rheumatological population. Although this comparison should be interpreted cautiously because the rheumatology outpatient cohort was not sex-matched and cannot be considered a healthy control population, the higher prevalence of cortical irregularities and calcifications among patients undergoing hemodialysis supports the hypothesis that chronic kidney disease-related mineral and bone disorder may contribute to TMJ structural remodeling.

Several complications of long-term dialysis were not specifically addressed in our design. Dialysis-related amyloidosis, driven by deposition of beta-2-microglobulin, is a major complication of long-term dialysis that can cause subchondral bone erosion [48]; we did not specifically investigate this process, although condylar surface irregularities were more prevalent among patients treated for less than 30 months than among those treated for longer, a finding that merits further mechanistic study rather than a straightforward amyloidosis explanation, since amyloid deposition would be expected to accumulate with, rather than inversely relate to, dialysis duration. This unexpected, inverse association should also be considered in light of the single comparison that reached significance among the many performed, and may partly reflect a chance finding; alternative, non-mutually exclusive explanations include survivor bias (patients who accrue condylar damage early may be selectively represented among those with shorter observed vintage, for example, through differential mortality or transplantation among longer-treated patients), referral or selection bias in a single-center consecutive sample, and residual confounding by unmeasured variables such as pre-dialysis CKD duration, prior parathyroid status, or bone-active medication use. We cannot distinguish between these possibilities in the present cross-sectional design.

CKD-MBD is now understood to significantly alter maxillary and mandibular bone structure, with oral manifestations ranging from tooth mobility and malocclusion to hypoplasia and pulp stones; radiographically, patients often show bone demineralization, cortical thinning and reduced trabeculation, increasing the risk of giant cell lesions, spontaneous or procedural jaw fracture, and impaired healing after dental extraction [49]. In hemodialysis patients with hyperparathyroidism, marked osteoclastic bone resorption has been observed specifically at tendon insertion sites [50]. Given the high prevalence of oral and periodontal disease among dialysis patients, closer collaboration between dental professionals and dialysis teams is warranted to ensure adequate follow-up of oral and craniofacial disease in this population [51]. Contemporary reviews of CKD-MBD pathophysiology continue to emphasize that mineral, hormonal and immune-mediated pathways act in concert on the skeleton, reinforcing the biological plausibility of TMJ involvement as part of this systemic process rather than an isolated dental finding [21,23].

### Limitations

Although this topic represents a relatively novel contribution to the literature on complications of end-stage kidney disease, several limitations should be acknowledged.

First, the study is limited by a relatively small, single-center sample, and the sample size was not derived from a formal power calculation for a pre-specified primary outcome. Moreover, the difference in age and sex distribution between the hemodialysis cohort and the healthy control group and the rheumatological patients require cautious interpretation. However, our study was designed as a descriptive, hypothesis-generating survey.

Second, the cross-sectional design allowed only a single evaluation of the TMJ, without longitudinal follow-up to assess progression.

Third, the absence of a non-dialysis control group limits our ability to attribute the observed TMJ changes specifically to the uremic and dialysis-related environment rather than to age-related degeneration alone; future studies should include age- and sex-matched controls, and longitudinal cohorts, to determine whether these largely subclinical abnormalities progress to symptomatic disease over time.

Fourth, several established risk factors for TMJ abnormalities were not systematically assessed, including previous TMJ trauma, smoking or diabetes severity; their absence limits our ability to account for potential confounding, and future studies should collect these variables.

Fifth, symptom status was based on the absence of spontaneously reported complaints rather than a validated TMD questionnaire, which may have underestimated true symptomatic prevalence.

Sixth, TMJ US is inherently operator-dependent, and although examinations were performed by a single experienced examiner to minimize inter-observer variability, we did not formally assess intra-observer reproducibility or compare TMJ US findings against MRI as a reference standard in this cohort.

Finally, as a single-center Italian cohort of predominantly elderly patients, the generalizability of these findings to other populations, dialysis modalities, or younger patients with ESRD is limited.

## 5. Conclusions

To the best of our knowledge, this is the first study to systematically evaluate TMJ abnormalities by ultrasound in an adult hemodialysis population. We found a substantial burden of subclinical TMJ abnormalities in this population, none of which were spontaneously reported by patients and none of which were assessed against a validated TMD questionnaire; the abnormalities encompassed both inflammatory and degenerative alterations, with degenerative findings clearly predominating. This cross-sectional study supports the feasibility of TMJ US in this population and describes the pattern of these largely subclinical findings, but, in the absence of an age- and sex-matched control group, it does not establish that this burden exceeds what would be expected from age-related degeneration alone, nor does it demonstrate a clinical benefit from routine screening. Notably, TMJ degeneration did not correlate with age, dialysis vintage, comorbidity burden, nutritional status or classical biochemical markers of bone metabolism on univariate analysis; given the large number of comparisons performed, these negative findings and the single positive association observed (condylar/cortical irregularity with shorter dialysis vintage) should be considered exploratory and hypothesis-generating rather than confirmatory. These results nonetheless underscore the complex, multifactorial interplay between the TMJ and end-stage renal disease and suggest that the joint may be a susceptible, if under-recognized, target within the broader landscape of systemic metabolic derangement in uremia. These findings warrant replication in larger, multicenter, longitudinal cohorts, ideally including matched controls, to confirm their generalizability and to clarify the natural history of subclinical TMJ involvement in CKD.

## Figures and Tables

**Table 1:** Demographic and clinical parameters of the 100 haemodialysis patients investigated.

	Value (*n* = 100)
Male/Female, *n* (%)	65 (65)/35 (35)
Age (years)	71.6 ± 13.1
Hemodialysis vintage (months)	50.6 ± 96.6
Serum calcium (mg/dL)	9.1 ± 0.5
ln (serum calcium)	2.2 ± 0.06
Serum phosphorus (mg/dL)	5.7 ± 1.4
ln (serum phosphorus)	1.7 ± 0.2
Parathyroid hormone (pg/mL)	218 ± 156
ln (parathyroid hormone)	5.0 ± 0.9
Charlson Comorbidity Index (CCI)	4.1 ± 1.9
CONUT score	3.4 ± 2.0

**Table 2:** Ultrasound findings of the temporomandibular joint examination (*n* = 100).

Findings	*n* (%)
Inflammatory findings	
Joint effusion	16 (16)
Synovial hypertrophy	15 (15)
Power Doppler (PD) signal	1 (1)
Cartilage changes	15 (15)
Degenerative findings	
Structural abnormalities	0 (0)
Condylar irregularities	33 (33)
Enthesophytes	25 (25)
Erosions	0 (0)
Calcifications	36 (36)

**Table 3:** Comparison of temporomandibular joint ultrasonographic findings between healthy controls and patients receiving chronic hemodialysis.

Variable	Healthy Controls (*n* = 40)	Hemodialysis Patients (*n* = 100)	*p*
Female sex	70.0%	35.0%	0.0003
Joint effusion (VA)	2.5%	16.0%	0.041
VA monolateral	2.5%	11.0%	0.179
VA bilateral	0.0%	6.0%	0.183
Synovial hypertrophy (IS)	2.5%	15.0%	0.040
IS monolateral	2.5%	14.0%	0.067
IS bilateral	0.0%	1.0%	1.000
Erosions	5.0%	0.0%	0.080
Erosions, right	2.5%	0.0%	0.286
Erosions, left	2.5%	0.0%	0.286
Power Doppler (PD) signal	0.0%	1.0%	1.000
PD signal, monolateral	0.0%	1.0%	1.000
PD signal, bilateral	0.0%	0.0%	1.000
Reduced cartilage thickness (RS)	20.0%	15.0%	0.460
RS monolateral	15.0%	11.0%	0.570
RS bilateral	5.0%	4.0%	1.000
Structural inhomogeneity (DE)	0.0%	0.0%	1.000
DE monolateral	0.0%	0.0%	1.000
DE bilateral	0.0%	0.0%	1.000
Cortical/condylar irregularity (ICO)	2.5%	33.0%	<0.0001
ICO monolateral	2.5%	23.0%	0.0024
ICO bilateral	0.0%	9.0%	0.060
Enthesophytes	0.0%	25.0%	0.0001
Enthesophytes, monolateral	0.0%	24.0%	0.0003
Enthesophytes, bilateral	0.0%	2.0%	1.000
Calcifications, monolateral	0.0%	26.0%	0.0001
Calcifications, bilateral	0.0%	10.0%	0.062
Disc dislocation, right	0.0%	2.0%	1.000
Disc dislocation, left	2.5%	5.0%	0.674

**Table 4:** Comparison between individuals younger than 73 years and those aged 73 years or older.

	Age < 73 y (*n* = 50)	Age ≥ 73 y (*n* = 50)	*p*
Female, n (%)	13 (26)	22 (44)	0.093
Male, n (%)	37 (74)	28 (56)	0.093
Age (years)	61.2 ± 9.8	82.1 ± 5.3	<0.0001
Hemodialysis vintage (months)	58.3 ± 125.6	42.9 ± 54.5	0.923
Hemodialysis vintage ≥ 30 months, *n* (%)	26 (52)	26 (52)	0.920
Serum calcium (mg/dL)	9.1 ± 0.6	9.0 ± 0.6	0.414
Serum phosphorus (mg/dL)	5.9 ± 1.6	5.5 ± 1.1	0.106
Parathyroid hormone (pg/mL)	223 ± 163	212 ± 151	0.717
CCI	3.06 ± 1.64	5.22 ± 1.71	<0.001
CCI ≥ 4, *n* (%)	18 (36)	45 (90)	<0.0001
CONUT score	3.38 ± 2.25	3.50 ± 1.90	0.620
CONUT score ≥ 3, *n* (%)	33 (66)	35 (70)	0.830
TMJ US findings			
Joint effusion, *n* (%)	4 (8)	12 (24)	0.054
Synovial hypertrophy, *n* (%)	7 (14)	8 (16)	1.000
Cartilage changes, *n* (%)	7 (14)	8 (16)	1.000
Condylar irregularities, *n* (%)	16 (32)	17 (34)	1.000
Enthesophytes, *n* (%)	10 (20)	15 (30)	0.356
Calcifications, *n* (%)	16 (32)	20 (40)	0.532

CCI: Charlson Comorbidity Index.

**Table 5:** Comparison between individuals with a hemodialysis vintage of less than 30 months (mo) and those with hemodialysis vintage of 30 months or more.

	Time < 30 mo (*n* = 48)	Time ≥ 30 mo (*n* = 52)	*p*
Female, *n* (%)	13 (27.1)	22 (42.3)	0.143
Male, *n* (%)	35 (72.9)	30 (57.7)	0.143
Age (years)	72.2 ± 13.0	71.1 ± 13.2	0.475
Hemodialysis vintage (months)	14.2 ± 7.6	84.9 ± 125.2	<0.0001
Serum calcium (mg/dL)	8.9 ± 0.5	9.2 ± 0.5	0.046
Serum phosphorus (mg/dL)	5.5 ± 1.3	5.9 ± 1.4	0.164
Parathyroid hormone (pg/mL)	230 ± 180	206 ± 132	0.636
CCI	4.48 ± 2.20	3.83 ± 1.74	0.103
CCI ≥ 4, *n* (%)	31 (64.6)	32 (61.5)	0.837
CONUT score	3.65 ± 2.32	3.25 ± 1.82	0.344
CONUT score ≥ 3, *n* (%)	33 (68.8)	35 (67.3)	1.000
TMJ US findings			
Joint effusion, *n* (%)	9 (18.8)	7 (13.5)	0.588
Synovial hypertrophy, *n* (%)	4 (8.3)	11 (21.2)	0.095
Cartilage changes, *n* (%)	7 (14.6)	8 (15.4)	1.000
Condylar irregularities, *n* (%)	21 (43.8)	12 (23.1)	0.034
Enthesophytes, *n* (%)	11 (22.9)	14 (26.9)	0.818
Calcifications, *n* (%)	16 (33.3)	20 (38.5)	0.678

CCI: Charlson Comorbidity Index.

## Data Availability

The original contributions presented in this study are included in the article/Supplementary Materials. Further inquiries can be directed to the corresponding author.

## Supplementary Materials

The following supporting information can be downloaded at: https://www.mdpi.com/article/10.3390/medsci14040492/s1, Table S1: Definitions of ultrasonographic findings used for temporomandibular joints; Table S2: TMJ abnormalities and severity distribution; Table S3: Association between local temporomandibular joint risk factors and ultrasonographic abnormalities in patients undergoing chronic hemodialysis; Table S4: Comparison of temporomandibular joint ultrasonographic findings between patients undergoing chronic hemodialysis and an age-comparable rheumatology outpatient cohort.

## Abbreviations

The following abbreviations are used in this manuscript:

CCI	Charlson comorbidity index
CKD	Chronic kidney disease
CKD-MBD	Chronic kidney disease-mineral and bone disorder
CONUT	Controlling nutritional status
ESRD	End-stage renal disease
FGF23	Fibroblast growth factor 23
GUESS	Glasgow ultrasound enthesitis scoring system
MRI	Magnetic resonance imaging
PTH	Parathyroid hormone
PD	Power Doppler
SD	Standard deviation
TMJ	Temporomandibular joint
TMJ US	Temporomandibular joint ultrasound
