# Supplementary material for: Temporomandibular Joint Abnormalities in Hemodialysis Patients: A Cross-Sectional Ultrasonographic Study from a Single Center in Italy
Source: Med Sci (Basel). 2026 Aug 19;14(4):492. doi: 10.3390/medsci14040492 (PMC13515121; doi:10.3390/medsci14040492)
Supplement: Supplementary file 1 [file medsci-14-00492-s001.zip › medsci-4463380-supplementary.pdf]

**Supplementary Table S1.** *Definitions of ultrasonographic findings used for temporomandibular joints*

| Ultrasonographic findings | Definition / Assessment | Scoring | Notes / Abbreviations |
|---|---|---|---|
| Synovial hypertrophy | Presence of ≥1 red spot in synovial hypertrophy area | 0–3 (0=normal, 1=mild, 2=moderate, 3=marked) | Reflects synovial proliferation |
| Power Doppler signal (PD) | Synovial hypertrophy + PD signal | 0–3 (0=absent, 1=≤3 spots, 2=≥3 spots <50% area, 3=>50% area) | Semi-quantitative, Naredo score |
| Active synovitis | Synovial hypertrophy without PD signal | N/A | Indicator of ongoing inflammation |
| Inactive synovitis | Abnormal intra-articular hypo-/anechoic material, compressible, no PD signal | N/A | Chronic / non-active inflammation |
| Joint effusion | Discontinuity of cortical bone, seen in two perpendicular planes | 0–3 (0=normal, 1=mild, 2=moderate, 3=marked) | Semi-quantitative |
| Condylar erosion | Step-like bony prominence at joint margin | N/A | Structural damage |
| Osteophytes | Disruption, roughness, prominences, or depressions of cortical line | N/A | Degenerative feature |
| Cortical irregularity | Loss of rounded condylar contour | N/A | Structural change |
| . | Decreased distance capsule-condyle <1.4 mm | N/A | Degenerative / remodeling |
| Cartilage thickness reduction | Loss of normal hypo-/anechoic cartilage layer | N/A | Reference value from US literature |
| Cartilage echostructural abnormalities | Anterior displacement of disc at mouth closed, no reposition on opening | N/A | Structural change; no OMERACT standard for TMJ fibrocartilage (1-3) |
| Disc displacement without reduction | Condyle remains visible during dynamic opening (reduced mandibular motion) | N/A | Structural evaluation |
| Dynamic hypomobility | | N/A | Indicates restricted mobility |

*Abbreviations: DC/TMD = Diagnostic Criteria for Temporomandibular Disorders; N/A = not applicable; PD = Power Doppler; US = ultrasonography.*

**Supplementary Table S2**. TMJ abnormalities and severity distribution.

| **Abnormality** | **Right total** | **Right severity (G1 / G2 / G3)** | **Left total** | **Left severity (G1 / G2 / G3)** | **Right Only** | **Left Only** | **Bilateral** | **Total patients affected (%)** |
|---|---|---|---|---|---|---|---|---|
| Joint effusion | **11** | 8/1/2 | **12** | 7/2/10 | 5 | 6 | 5 | **16** |
| Synovial hypertrophy | **6** | 1/2/3 | 10 | 4/2/4 | 5 | 9 | 1 | **15** |
| Power Doppler signal | 1 | 1/0/0 | 0 | 0 | 1 | 0 | 0 | **1** |
| Cartilage changes | 6 | 4/0/2 | 5 | 4/0/1 | 6 | 5 | 4 | **15** |
| Structural abnormalities | **0** | 0 | **0** | 0 | 0 | 0 | 0 | **0** |
| Condylar irregularities | 12 | 11/0/1 | 11 | 8/1/2 | 12 | 11 | 10 | **33** |
| Enthesophytes | 13 | 9/2/2 | 11 | 6/5/0 | 13 | 11 | 1 | **25** |
| Erosions | **0** | 0 | **0** | 0 | 0 | 0 | 0 | **0** |
| Calcifications | 12 | 7/5/0 | 14 | 9/4/1 | 12 | 14 | 10 | **36** |

** Notes: G1 = Grade 1, G2 = Grade 2, G3 = Grade 3. Semiquantitative (Grade 1–3) grading of ultrasonographic inflammatory and structural temporomandibular joint abnormalities according to OMERACT-based definitions, where applicable.*

*N/A applies to parameters evaluated as binary presence/absence.*

**Supplementary Table S3**. Association between local temporomandibular joint risk factors and ultrasonographic abnormalities in patients undergoing chronic hemodialysis.

| Variable | Overall (n=100) | Any US abnormality | No US abnormality |
|---|---|---|---|
| Edentulism, n (%) | 8 | 2 (calcifications), 1 (synovial hypertrophy) | 5 |
| Malocclusion, n (%) | 1 | 0 | 1 |
| Bruxism, n (%) | 1 | 1 (calcifications) | 0 |

**Supplementary Table S4**. Comparison of temporomandibular joint ultrasonographic findings between patients undergoing chronic hemodialysis and an age-comparable rheumatology outpatient cohort.

| Variable | Hemodialysis patients (n = 100) | Rheumatology outpatient controls (n = 46) | *p* value |
|---|---|---|---|
| **Age, years** | 71.6 ± 13.1 | 70.0 ± 9.5 | 0.446 |
| Female sex, n (%) | 35 (35.0) | 35 (76.1) | <0.0001 |
| Male sex, n (%) | 65 (65.0) | 11 (23.9) | — |
| **Ultrasonographic findings** | | | |
| Joint effusion, n (%) | 16 (16.0) | 10 (21.7) | 0.485 |
| Synovial hypertrophy, n (%) | 15 (15.0) | 2 (4.3) | 0.093 |
| Bone erosions, n (%) | 0 (0.0) | 1 (2.2) | 0.315 |
| Positive Power Doppler signal, n (%) | 1 (1.0) | 2 (4.3) | 0.234 |

| Variable | Hemodialysis patients (n = 100) | Rheumatology outpatient controls (n = 46) | *p* value |
|---|---|---|---|
| Cartilage thinning, n (%) | 15 (15.0) | 11 (23.9) | 0.244 |
| Structural cartilage heterogeneity, n (%) | 0 (0.0) | 3 (6.5) | 0.030 |
| Cortical irregularities, n (%) | 33 (33.0) | 3 (6.5) | <0.0001 |
| Enthesophytes, n (%) | 25 (25.0) | 10 (21.7) | 0.835 |
| Calcifications, n (%) | 36 (36.0) | 3 (6.5) | <0.0001 |
